# Molecular Dynamics Study on Silane Coupling Agent Grafting to Optimize the Interfacial Microstructure and Physical Properties of Polyimide/Nano-Si_3_N_4_ Composites

**DOI:** 10.3390/ma18184425

**Published:** 2025-09-22

**Authors:** Qikun Yang, Jinxin Huang, Li Zhang, Nurbek N. Kurbonov, Shengrui Zhou

**Affiliations:** 1 School of Electrical Engineering, Shandong University, Jinan 250061, China; 202314668@mail.sdu.edu.cn (Q.Y.); zhleee@sdu.edu.cn (L.Z.);; 2 School of Continuing Education, Shandong University, Jinan 250100, China; 3 Faculty of Electrical Power Engineering, Tashkent State Technical University, Tashkent 100095, Uzbekistan; nurbek.kurbonov.96@gmail.com

**Keywords:** polymer composites, silane coupling agent, microstructures, thermophysical properties, molecular dynamics

## Abstract

Polyimide (PI) is widely used in aerospace, electronic packaging, and other fields due to its excellent dielectric and thermophysical properties. However, the performance of traditional PI materials under extreme conditions has become increasingly inadequate to meet the growing demands. To address this, this study designed a PI/Nano-Si_3_N_4_ advanced composite material and, based on molecular dynamics simulations, thoroughly explored the influence of silane coupling agents with different grafting densities on the interfacial microstructure and their correlation with the overall material’s physical properties. The results show that when the grafting density is 10%, the interfacial bonding of the PI/Nano-Si_3_N_4_ composite is optimized: non-bonded interaction energy increases by 18.4%, the number of hydrogen bonds increases by 32.5%, and the free volume fraction decreases to 18.13%. These changes significantly enhance the overall performance of the material, manifested by an increase of about 30 K in the glass transition temperature and a 49.5% improvement in thermal conductivity compared to pure PI. Furthermore, the system maintains high Young’s modulus and shear modulus in the temperature range of 300–700 K. The study reveals that silane coupling agents can effectively enhance the composite material’s overall performance by optimizing the interfacial structure and controlling the free volume, providing an efficient computational method for the design and performance prediction of advanced high-performance PI composites.

## 1. Introduction

Polyimide (PI) is a high-performance polymer characterized by a main chain containing imide rings. Due to its excellent high-temperature resistance, chemical corrosion resistance, and outstanding insulating and mechanical properties, PI is widely used in fields such as power electronics, the automotive industry, and semiconductor packaging [1,2,3,4]. With the ongoing upgrades in high-voltage transmission systems and the continuous development of aerospace technologies, traditional PI materials have become inadequate in meeting the demands of extreme conditions, particularly in terms of long-term thermal stability and mechanical properties across a wide temperature range [5,6]. To address this, researchers have focused on developing organic–inorganic hybrid PI composites by introducing nanofillers such as silica [7], alumina [8], boron nitride [9], and graphene [10], aiming to synergistically enhance the overall material properties. However, due to the large specific surface area and high surface energy of nanoparticles, they tend to agglomerate within the polymer matrix, leading to interface defects that significantly degrade the overall performance of the composite material [11,12,13,14]. To resolve this key issue, surface modification techniques have been proven to be an effective method for improving the filler–matrix interfacial compatibility [15]. This technology, through chemical grafting or physical coating methods, can regulate the surface characteristics of nanoparticles, thereby enhancing their interaction with the PI matrix. The relevant mechanisms and their effects on the composite material properties have become a research hotspot [16,17,18,19].

Silane coupling agents are a class of highly efficient interfacial modifiers. One end of their molecule binds to polymer chains via non-covalent or covalent interactions, while the alkoxy groups at the other end hydrolyze and covalently bond with hydroxyl groups on the surface of inorganic fillers, thereby constructing an “organic matrix–coupling agent–inorganic filler” bridging structure. This configuration markedly enhances interfacial adhesion and improves the overall thermal, mechanical, and electrical properties of the composite material [20]. Xiao C.G. et al. [21] compared the modification effects of three silane coupling agents—KH550, KH560, and KH570-in PI/SM composites—finding that KH550 produced the strongest interfacial bonding and the most pronounced improvements in thermal and mechanical performance. Liu L.Z. et al. [22] employed KH550, KH560, and AE3012 to modify the surface of nano-Al_2_O_3_, and reported that although AE3012 modification yielded the highest breakdown strength (290 kV/mm) in PI/Al_2_O_3_ hybrid films, the KH550-modified system exhibited tensile strength and elongation at break increases of 14.2% and 78.5%, respectively, over the unmodified system, demonstrating outstanding mechanical reinforcement. Chi H.M. et al. [23] prepared polyimide fibrous membranes incorporating KH550-treated SiO_2_ as the interfacial filler; FTIR and SEM analyses revealed significantly enhanced interfacial compatibility, and the composite paper containing 3 wt% KH550-SiO_2_ exhibited a reduced dielectric constant (2.32) and a peak dielectric strength of 85 kV/mm. More recently, P. Jayaseelan et al. [24] demonstrated that silane coupling treatment can substantially strengthen the interfacial bonding of nano-ZnO/polymer composites and improve their tribological properties, reinforcing the broad applicability of silane coupling agents, such as KH550, in systematically tuning interfacial compatibility. Despite these substantial achievements in enhancing macroscopic properties, most studies remain at the level of experimental characterization. Systematic and quantitative elucidation of the interfacial interaction mechanisms at the atomic/molecular scale and of the structure–property relationships at the microscopic level is still lacking, which, to some extent, constrains the precise design and optimization of high-performance polyimide composites.

In recent years, advanced modeling and simulation techniques have been increasingly employed to predict and optimize the properties of composite materials. A. Bifulco et al. [25] successfully applied machine learning to predict the combustion characteristics of multilayer polymer systems, demonstrating the synergistic advantages of combining data-driven modeling with simulation in materials design. At the atomic scale, molecular dynamics (MD) simulations, owing to their exceptional capability for dynamic analysis, have become a key approach for elucidating multiscale interfacial mechanisms in polymer–filler systems [26,27,28,29]. Liang M.F. et al. [30] used MD simulations to systematically investigate the effects of SiO_2_ nanoparticle size and morphology on the thermodynamic behavior of PI/SiO_2_ interfaces, revealing that the interfacial binding energy decays exponentially with increasing particle size, and that spherical fillers, compared with cylindrical or rod-like geometries, are more favorable for forming compact interfacial contacts with PI chains. Zhao Y.L. et al. [31] conducted a comparative MD study on the mechanisms by which three silane coupling agents regulate the properties of PI/SiO_2_ composites, finding that KH550 coupling agent synergistically enhances both the thickness and density of the interfacial transition layer, resulting in optimal interfacial adhesion performance. Nevertheless, existing computational studies have primarily focused on qualitative analyses of filler size and coupling agent type, while systematic atomic-scale investigations and in-depth elucidations of the quantitative structure–property relationships between coupling agent grafting density and interfacial characteristics remain scarce.

Nano-Si_3_N_4_, as a high-performance ceramic filler, exhibits superior high-temperature oxidation resistance and thermal shock resistance compared with conventional inorganic fillers [32,33,34,35]. Zhang Z.L. et al. [36] incorporated nano-Si_3_N_4_ at various mass fractions into fluorinated polyimide to prepare composite PI films, and experimental results confirmed that the introduction of Si_3_N_4_ significantly enhances the thermal stability and dielectric properties of the films. However, existing studies have primarily focused on macroscopic performance testing of PI/Si_3_N_4_ films, while the evolution of the interfacial structure in the composite system after Si_3_N_4_ incorporation and the microscopic mechanisms by which it influences material properties remain largely unexplored, particularly with respect to atomic-scale theoretical analysis. To address this scientific question, the present study employs MD simulations to construct a pure PI model, an unmodified PI/Si_3_N_4_ composite model, and four KH550-modified systems with grafting densities of 5%, 7%, 10%, and 14%. By calculating and analyzing key microstructural parameters such as interaction energy, radial distribution function, hydrogen bond number, and free volume fraction, along with macroscopic performance characterizations including thermal conductivity, glass transition temperature, and elastic modulus, the study deeply explored the regulation of surface grafting density on the interface structure and properties of PI/Si_3_N_4_ composites. It established a quantitative correlation mechanism between microstructural parameters and macroscopic performance. The study further clarified the microscopic mechanism by which the KH550 coupling agent enhances the interfacial compatibility of PI/Si_3_N_4_ and identified the optimal grafting density, providing important theoretical guidance for surface modification of nanofillers and the design of high-performance PI composites.

## 2. Methods and Models

**Figure 1:**
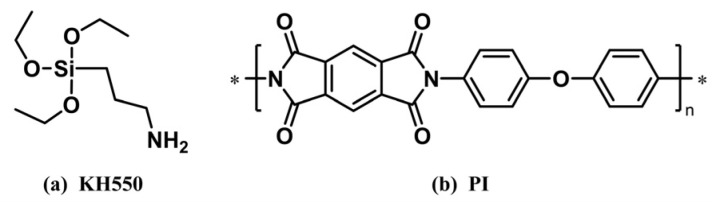
Chemical structural formulas of KH550 and PI. (**a**) KH550 silane coupling agent; (**b**) repeating unit of Kapton-type PI. The asterisk (*) indicates the reactive site in the PI repeating unit.

**Figure 2:**
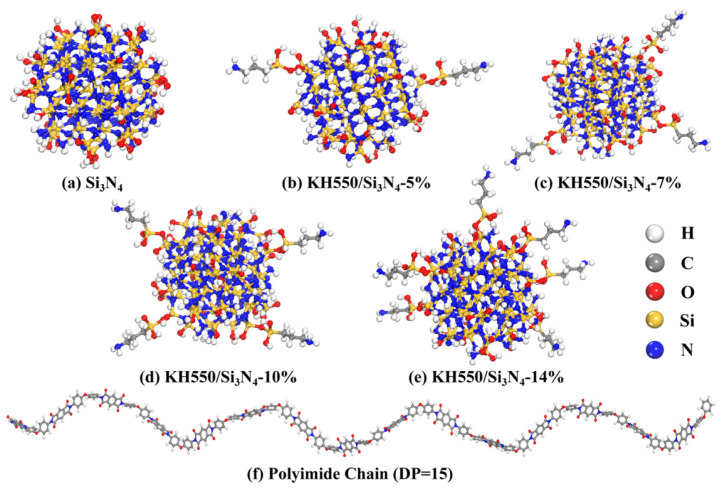
Silicon nitride nanoparticles at varying grafting densities and PI molecular chains. (**a**) pristine Si_3_N_4_; (**b**–**e**) Si_3_N_4_ with 5%, 7%, 10%, and 14% KH550 grafting densities, respectively; (**f**) PI molecular chain with a degree of polymerization of 15.

**Figure 3:**
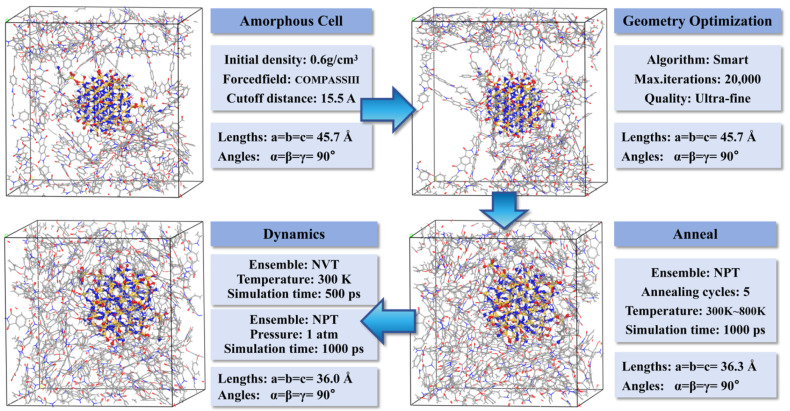
Procedure for unit cell optimization.

**Figure 4:**
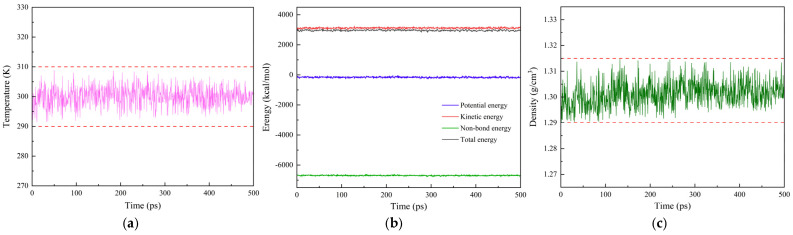
Criteria for system equilibrium state (taking pure PI system as an example). (**a**) Temperature fluctuation curve; (**b**) energy fluctuation curve; (**c**) density fluctuation curve.

**Figure 5:**
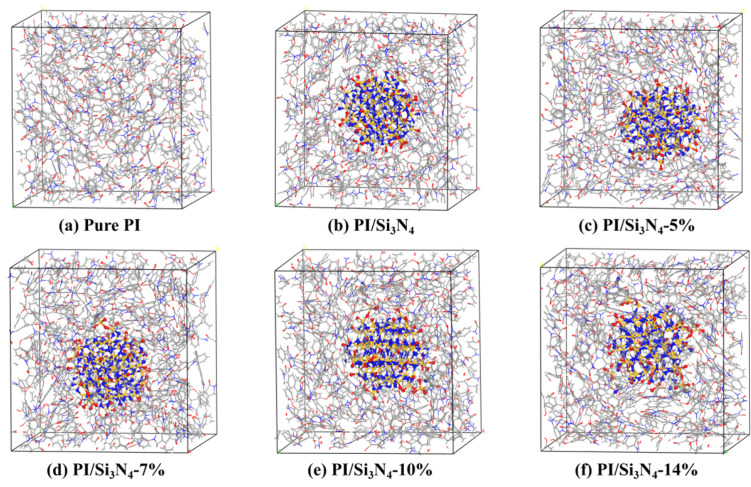
Stable unit cell models for all systems. (**a**) pure PI; (**b**) PI/pristine Si_3_N_4_; (**c**–**f**) PI/Si_3_N_4_ with 5%, 7%, 10%, and 14% KH550 grafting.

To systematically study the effects of nano-silicon nitride doping and variations in surface grafting density on the structure and properties of PI composites, six different simulation systems were established using the Materials Studio 2023 software. These included pure PI, unmodified silicon nitride, and PI/Si_3_N_4_-KH550 composite models with grafting densities of 5%, 7%, 10%, and 14%, respectively. The specific modeling process is as follows:

(1) An amorphous Si_3_N_4_ nanocluster sphere with an 8 Å radius was generated from the β-Si_3_N_4_ unit cell. To mimic oxidation of high surface area Si_3_N_4_ nanoparticles under realistic conditions, surface Si-H bonds on the cleaved cluster were replaced with Si-O bonds and subsequently saturated with hydrogen atoms, yielding 42 hydroxyl groups. A subset of these hydroxyls was then randomly chosen as grafting sites—spatially separated to prevent clustering—for attachment of 0, 2, 3, 4, and 6 KH550 silane coupling–agent chains (chemical structure shown in Figure 1a), corresponding to grafting densities of 0%, 5%, 7%, 10%, and 14%, respectively. All models underwent geometry optimization using the COMPASS III force field until energy convergence, with energy and force tolerances of 10^−4^ kcal·mol^−1^ and 0.005 kcal·mol^−1^·Å^−1^, respectively, and atomic charges were determined by the QEq method. The optimized structures are shown in Figure 2a–e.

(2) Guided by engineering application requirements, the model adopts a Kapton-type PI composed of pyromellitic dianhydride (PMDA) and 4,4′-oxydianiline (ODA) monomers [37,38], as shown in Figure 1b. Prior studies indicate that when the degree of polymerization (DP) is 10–15, para-type PI chains effectively suppress end-group artifacts while faithfully reproducing bulk thermodynamic properties, thereby achieving an optimal balance between computational accuracy and efficiency [39,40,41]. Accordingly, a para-type PI chain with DP = 15 was constructed as the matrix model. Chain geometries were optimized to energy convergence using the COMPASS III force field, and partial charges were assigned using the QEq method. The optimized molecular structure is shown in Figure 2f.

(3) The optimized PI molecular chains were filled into a periodic box using the Amorphous Cell module. To avoid molecular chain end interactions caused by spatial congestion, an initial density of 0.6 g/cm^3^ and six chains were set. Subsequently, Si_3_N_4_ nanoparticles with grafting densities of 0%, 5%, 7%, 10%, and 14% were introduced, generating the initial models for six systems: pure PI, PI/Si_3_N_4_, PI/Si_3_N_4_-5%, PI/Si_3_N_4_-7%, PI/Si_3_N_4_-10%, and PI/Si_3_N_4_-14%.
(4) Due to the instability of the initial unit cell structure, energy optimization was necessary to eliminate residual stress and bring the hole distribution of the unit cell closer to that of the actual material. The optimization process is shown in Figure 3. First, a “Smart” algorithm was used for 20,000 steps of geometric optimization with a step size of 1 fs. Subsequently, the model underwent annealing treatment in the temperature range of 300 K to 800 K, lasting for five cycles, with a total duration of 1000 ps. Next, the configuration with the lowest potential energy during the annealing process was selected for MD simulations, followed by 500 ps equilibration using the NVT ensemble (with an Andersen thermostat) and 1000 ps relaxation using the NPT ensemble (at 1 atm pressure, with a Berendsen barostat). During this process, electrostatic interactions and van der Waals forces were calculated using the Ewald and Atom-based methods, respectively.

(5) There are typically two criteria for determining whether the system has reached a balanced state: first, the temperature fluctuation range of the system should be controlled within 15 K; second, the system’s energy should fluctuate within a fixed range. Taking the pure PI system as an example, the temperature, energy, and density fluctuation curves are shown in Figure 4. As the relaxation time increases, the temperature and energy of the pure PI system gradually reach equilibrium. By observing the last 200 ps of the relaxation process, the maximum temperature fluctuation is 6.77 K, and the energy fluctuation range is within 1.5%, indicating that the system has reached equilibrium. Additionally, the density fluctuation range is 1.302 ± 0.88% g/cm^3^, which is consistent with the simulated or experimental PI density values reported in the literature (1.28–1.38 g/cm^3^) [42,43,44], confirming that the model reasonably represents the target system and meets the requirements for subsequent research. The final stable models of all systems are shown in Figure 5.

(6) To obtain configurations at different temperatures (300 K to 800 K) for subsequent studies, the model in Figure 5 was first subjected to an NVT ensemble simulation at 800 K for 500 ps to allow the molecular chains to fully relax and adapt to the target temperature. Then, a stepwise cooling procedure was implemented with a 50 K interval (from 800 K to 300 K, across 11 stages), and each temperature point underwent a 500 ps NPT ensemble equilibration simulation. The last 150 ps of the trajectory were extracted as the equilibration phase, with one frame output every 2.5 ps, yielding a total of 60 configurations. To ensure data reliability and quantify statistical uncertainty, subsequent results for interfacial binding energy, glass transition temperature, and thermal conductivity were obtained by averaging over these 60 configurations, and the corresponding standard deviations were presented as error bars in the figures.

## 3. Results and Discussion

### 3.1. Interaction Energy Analysis

The interaction energy between nanoparticles and the polymer matrix serves as a critical indicator for evaluating the interfacial bonding strength of composites. Stronger interfacial bonding is beneficial for enhancing both the thermal and mechanical properties of the material. In the composite models constructed in this study, the para-type PI chains lack active functional groups capable of covalently reacting with the amino termini of KH550; therefore, the interfacial bonding in the PI/Si_3_N_4_ systems is predominantly governed by non-covalent interactions, including van der Waals forces and electrostatic interactions. To quantitatively assess the interfacial strength, the non-bonded interaction energy (*E_int_*) was introduced, where a more negative value indicates stronger PI/Si_3_N_4_ interactions and improved interfacial compatibility. The specific calculation formula is given as follows:

(1) $$E_{\mathrm{int}\;}=E_{\mathrm{PI}/{\mathrm{Si}}_{3}N_{4}}-E_{\mathrm{PI}}-E_{{\mathrm{Si}}_{3}N_{4}}$$

(2) $$E_{\mathrm{Binding}\;}=-E_{\mathrm{int}\;}$$

where $E_{\mathrm{PI}}$ and $E_{{\mathrm{Si}}_{3}N_{4}}$ are the molar internal energies of PI and nano-Si_3_N_4_ in the composite model, and $E_{\mathrm{Binding}\;}$ is the interfacial binding energy, defined as the negative value of the interaction energy.

**Figure 6:**
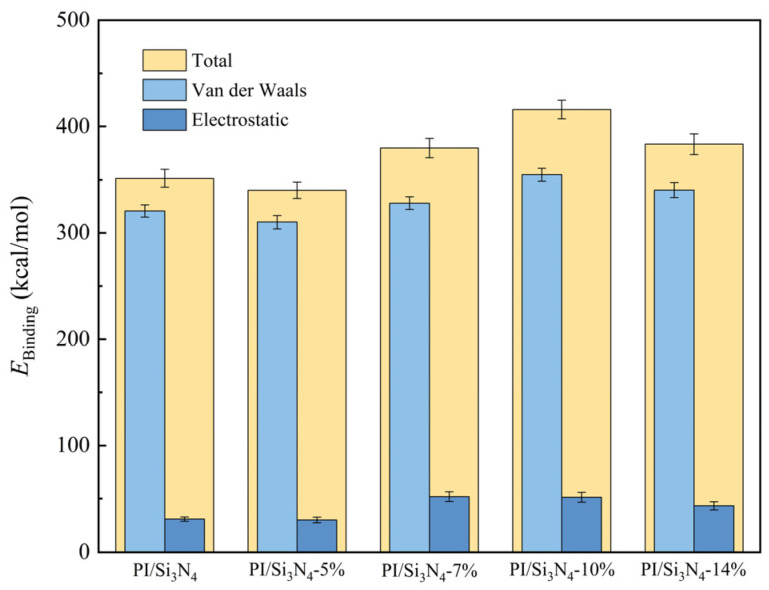
Interfacial binding energy between nanoparticles and PI at 300 K.

As shown in Figure 6, the interfacial binding energy (*E_binding_*) and its component contributions were calculated for each composite system. The results reveal that modification with the KH550 silane coupling agent markedly enhances the interactions between nanoparticles and the PI matrix. When the grafting ratio increases from 5% to 10%, *E_binding_* rises continuously, reaching its maximum at 10%, which is 18.4% higher than that of the unmodified system. However, further increasing the grafting ratio to 14% leads to a decline in *E_binding_*. In the 10% grafting system, the synergistic reinforcement of van der Waals forces and electrostatic interactions is most pronounced, resulting in optimal interfacial compatibility. By contrast, the reductions in *E_binding_* observed for the 5% low-grafting and 14% high-grafting systems are primarily attributed to the fact that van der Waals forces—being the dominant component of the binding energy—are sensitive to the interfacial spacing between polymer chains and inorganic fillers; variations in this spacing induced by different grafting densities ultimately weaken the interfacial bonding strength [45].

To verify the above hypothesis, the variation in coupling agent length (i.e., interfacial spacing) at different grafting ratios was characterized by measuring the distance between the terminal atom of the silane coupling agent and the Si atom, as summarized in Table 1. At the lower grafting density of 5%, KH550 molecules are sparsely distributed, and the chain segments remain largely independent, resulting in an incomplete coupling agent coating on the nanoparticle surface. This incomplete coverage not only fails to effectively shield the particle surface but also limits sufficient interaction with the matrix, leading to insufficient interfacial contact, an enlarged interfacial gap, and consequently weakened van der Waals interactions between the polymer and filler. With increasing grafting density, the surface coverage of KH550 on nanoparticles improves, and steric repulsion among coupling agent molecules intensifies. To minimize the system energy, the chain segments progressively bend and fold [31], thereby reducing the interfacial spacing and strengthening van der Waals forces. However, when the grafting ratio exceeds 10%, excessive coupling agents accumulate on the Si_3_N_4_ surface, forming a dense adsorbed layer that hinders direct contact between the PI matrix and the filler, leading to a decline in van der Waals forces. Furthermore, conformational changes in the coupling agents can also influence the electrostatic component of the binding energy. Electrostatic interactions are closely related to functional groups [46]; the amino groups at the termini of KH550 can effectively enhance electrostatic attraction between PI molecules and the Si_3_N_4_ surface. Nevertheless, at high grafting ratios, the dense adsorbed layer formed blocks the active sites on the Si_3_N_4_ surface, suppressing hydrogen bond formation at the interface and thereby reducing the electrostatic contribution. These results indicate that an appropriate amount of silane coupling agent grafting facilitates the enhancement of interfacial binding energy, whereas excessive grafting suppresses interactions between PI and Si_3_N_4_.

### 3.2. Free Volume Fraction Analysis

The total internal volume of polymer materials consists of two parts: the occupied volume and the free volume [47]. The occupied volume refers to the space occupied by the molecular chains of the material, while the free volume refers to the space in which the molecular chains can move freely, typically arising from the voids formed by the disordered packing of atoms. Considering the differences in the total volume of each system, free volume fraction (FFV) is used as an evaluation index for comparing the relative content of free volume. The specific calculation method is shown in Equation (3):

(3) $$FFV=\frac{V_{\mathrm{Free}\;}}{V_{\mathrm{Free}\;}+V_{\mathrm{Occupied}\;}}\times 100\%$$

where *FFV* represents the free volume fraction, $V_{\mathrm{Free}\;}$ represents the free volume of the system, and $V_{\mathrm{Occupied}\;}$ represents the volume occupied by the system.

In this study, the hard-sphere probe method was used to create a Connolly surface in Materials Studio software. The FFV of each system was measured using a spherical probe with a diameter of 0.1 nm, and the results are shown in Table 2. The FFV of the pure PI system was found to be 21.33%, which is in good agreement with the value reported in the literature [48,49], validating the reliability of the model. The study indicates that the Si_3_N_4_ nanoparticles occupy part of the free volume, restricting the movement of PI molecular chains and resulting in a lower FFV for the composite system compared to pure PI. At a 5% grafting density, the KH550 molecules are sparsely distributed on the Si_3_N_4_ surface, leading to an incomplete coating layer. Such discontinuous interfacial coverage introduces additional voids or structural defects at the interface, resulting in a higher FFV than that of the unmodified system. As the grafting density increases, the coupling agent molecules gradually bend and fill the interfacial voids, encouraging the PI molecular chains to aggregate on the surface of the nanoparticles, significantly compressing the free volume. However, when the grafting density exceeds 10%, the excessive accumulation of coupling agents on the particle surface forms defects such as micropores, while also shielding some of the active sites on the Si_3_N_4_ surface, reducing the restriction on PI molecular chains and causing the FFV to rise again.

**Figure 7:**
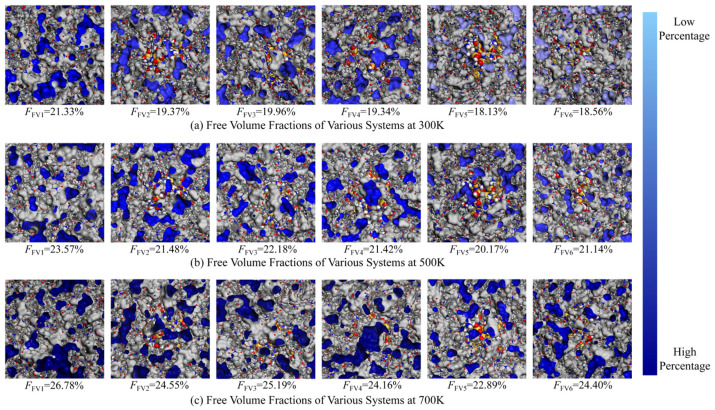
Cross-sectional distribution of the free volume for each system at different temperatures. (**a**) systems at 300 K; (**b**) systems at 500 K; (**c**) systems at 700 K.

**Figure 8:**
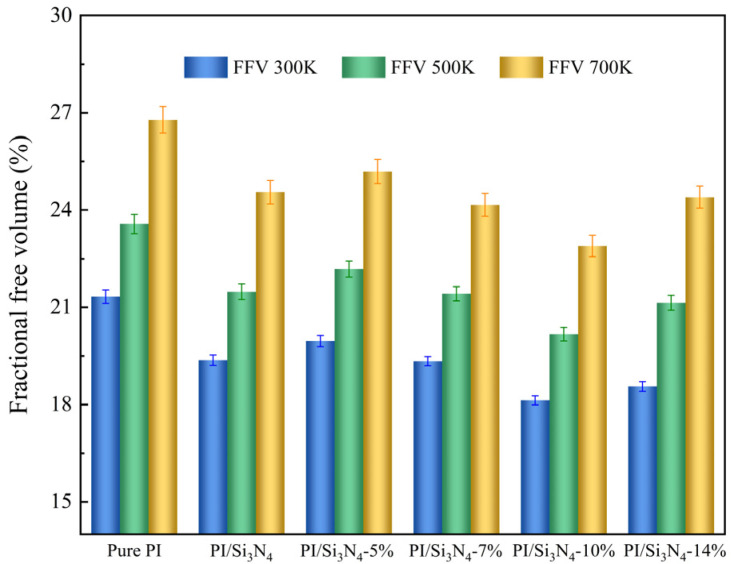
Fractional free volume of each system at different temperatures.

Figure 7 and Figure 8 show the distribution characteristics of the internal free volume and the FFV calculation results at different temperatures, where subscripts 1–6 correspond to the pure PI and PI/Si_3_N_4_ composite systems with grafting densities of 0%, 5%, 7%, 10%, and 14%, respectively. The blue surface represents the cross-section of the free volume, with the color intensity being proportional to the FFV. As the temperature increases from 300 K to 700 K, the color of the free volume cross-sections deepens, and small pores aggregate into larger ones, with their sizes significantly increasing. This indicates that high temperatures exacerbate interfacial defects, increasing the migration space for molecular chains, which negatively impacts the material properties. At high temperatures, the FFV of the composite systems is generally lower than that of pure PI because the rigid Si_3_N_4_ skeleton restricts the thermal expansion of the matrix, thereby reducing the free volume. Notably, in the composite system with a 10% grafting density, the FFV remains the lowest across all temperatures, indicating the best high-temperature stability. Combining with previous studies, it is evident that at a 10% grafting density, the coupling agent maintains its structural integrity at high temperatures, preserving interfacial bonding strength and effectively suppressing the volume expansion caused by thermal expansion. However, at a 14% grafting density, the excessive coupling agent leads to interfacial instability, and the intense thermal motion of the coupling agent layer promotes abnormal growth of the free volume. Therefore, a moderate grafting density of coupling agents can effectively alleviate the increase in thermal-induced free volume and enhance the thermal stability of the composite material.

### 3.3. Radial Distribution Function Analysis

**Figure 9:**
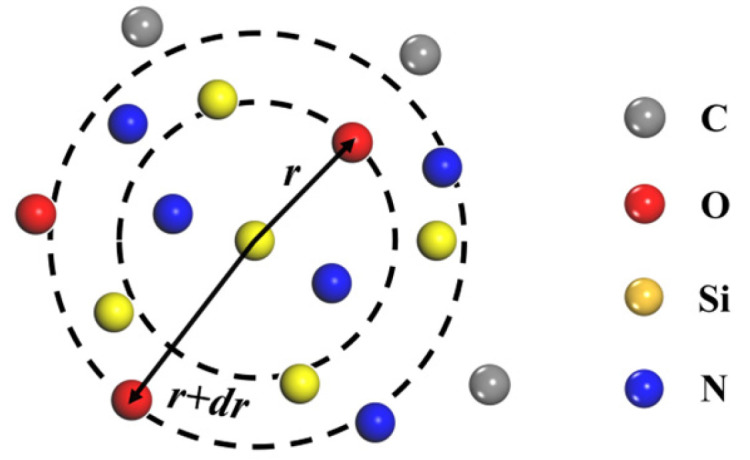
RDF schematic diagram.

The radial distribution function (RDF) is a characteristic physical quantity that describes the microstructure of materials. It is defined as the ratio of the probability density of finding another atom in a region at a distance *r* from a given atom to the average distribution density. The peak intensity directly reflects the strength of the interactions between atoms. The calculation formula is as follows:

(4) $$g(r)=\frac{dN}{\rho 4\pi r^{2}dr}$$

where dN is the number of atoms located within the spherical shell between radii *r* and r + dr from the reference atom, and ρ is the system density. A schematic illustration is provided in Figure 9.

**Figure 10:**
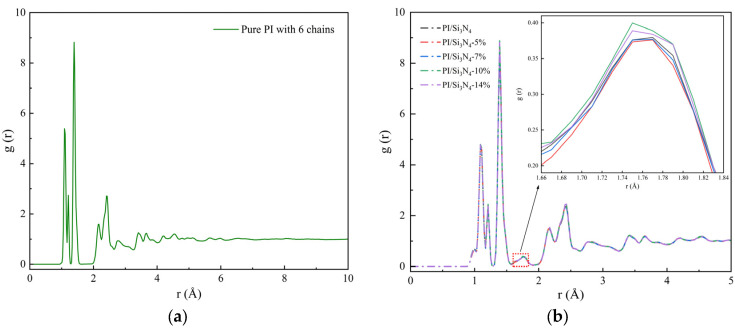
Average intramolecular radial distribution function, with an enlarged view in the inset from 1.66 Å to 1.84 Å. (**a**) Pure PI; (**b**) PI/Si_3_N_4_ composite systems with different grafting ratios.

Figure 10 shows the RDF curves of all atoms in each system. Both pure PI and PI/Si_3_N_4_ composite systems exhibit several similar characteristic peaks within the range of *r* < 4 Å, representing the short-range ordered structure of the system. For *r* > 5 Å, *g*(*r*) approaches a value of 1, consistent with the typical amorphous characteristics of polyimide [50]. Compared to pure PI (Figure 10a), the composite systems (Figure 10b) show an additional broad peak in the 1.65–1.85 Å range, indicating that an inorganic–organic interface forms a hydrogen-bond-dominated network structure, with the peak intensity reflecting the network density and order of the hydrogen bonds. The zoomed-in view shows that the *g*(*r*) peak is highest in the 10% grafting density system, followed by 14%, while the peaks for the 5%, 7%, and unmodified systems are significantly weaker, suggesting the existence of a critical threshold for grafting density. At low grafting densities, the coupling agents are sparsely distributed and cannot percolate to form a continuous hydrogen bond network, so the strengthening of interatomic interactions is limited. With increasing grafting density, the coupling agents progressively passivate interfacial defects and reinforce the interphase network, leading to denser packing of chain segments near the particle surface; correspondingly, short-range order is markedly enhanced in the 10% system. Once the grafting density exceeds the critical value, excess coupling agents introduce conformational crowding and interfacial disorder, causing a slight decrease in *g*(*r*) for the 14% system. Taken together, the average intramolecular RDF exhibits a nonlinear response to the Si_3_N_4_ grafting density of the silane coupling agent; a 10% grafting density represents the critical point for optimizing the interfacial network in polyimide nanocomposites. Below this threshold, the interphase strengthening increases with grafting density, whereas above it, the effect gradually diminishes due to interfacial instabilities.

**Figure 11:**
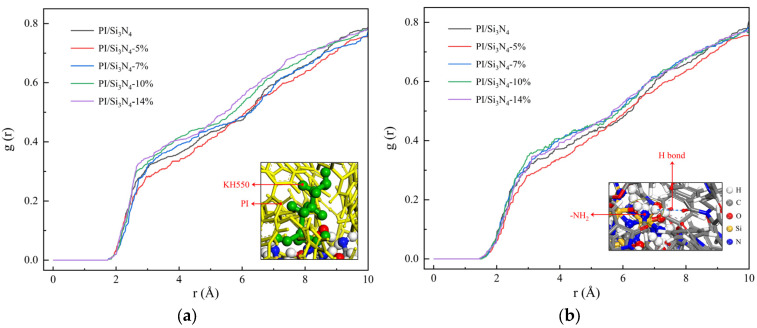
Average radial distribution function between nanoparticles and PI molecules. (**a**) At 300 K; (**b**) at 500 K.

To elucidate the effect of grafting density on the interfacial structure of the composite system at different temperatures, RDF between Si_3_N_4_ and hydrogen atoms in the PI chains was calculated at 300 K and 500 K, as shown in Figure 11. Higher RDF peaks indicate more pronounced accumulation of PI chains at the nanoparticle surface within the corresponding radial range. At 300 K, the high-grafting-density Si_3_N_4_ exhibited significantly higher RDF values in the 3–5 Å range compared to the ungrafted system, demonstrating an increased local packing density of PI chains around the nanoparticles. This enhancement arises because KH550 grafted onto the Si_3_N_4_ surface anchors PI chains via hydrogen bonding, and its polymer segments fill matrix voids, enriching H atoms in the interfacial region and strengthening nonbonded interactions. At 500 K, the high-grafting-density system still maintained elevated H-atom distribution probabilities, confirming the excellent thermal stability of the hydrogen bond network built by the coupling agent. Although this study employs a single-particle model, making it difficult to directly simulate particle–particle aggregation behavior, the RDF analysis results still indirectly confirm that graft modification facilitates dispersion and suppresses aggregation: an increase in RDF peak height indicates tighter local packing of PI chains around nanoparticles, suggesting that silane coupling agents improve interfacial compatibility and effectively isolate particles both energetically and spatially, thereby reducing the tendency for aggregation within the system.

### 3.4. Intermolecular Hydrogen Bond Analysis

The hydrogen bond network significantly influences the melting point, boiling point, and molecular conformation of polymeric materials. Its topological structure is not only a key factor in maintaining the mechanical strength of the polymer but also determines the thermal stability of the material. In this study, the intermolecular hydrogen bonding in each model system was analyzed. The atomic-scale structure of Si_3_N_4_ is highlighted using a ball-and-stick model, and the PI chains are simplified into a single-stick mode to facilitate the observation of the interfacial bonding characteristics.

**Figure 12:**
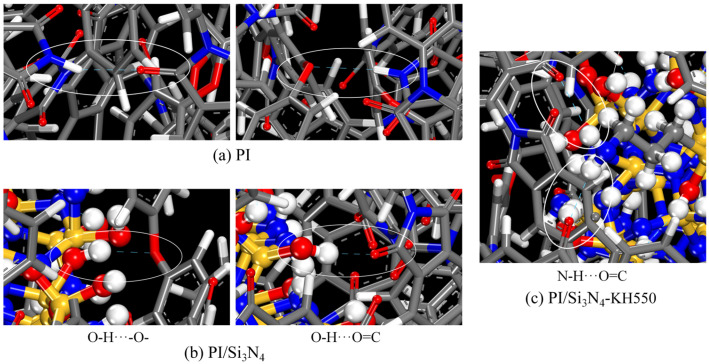
Intermolecular hydrogen bond composition in different systems. (**a**) PI; (**b**) PI/Si_3_N_4_; (**c**) PI/Si_3_N_4_–KH550.

As shown in Figure 12, in the unmodified PI/Si_3_N_4_ system, hydrogen bonds primarily arise from the coordination between the hydroxyl hydrogen (-OH) on the Si_3_N_4_ surface and the carbonyl oxygen (C = O) of the PI, with only a small amount forming between the hydroxyl and ether oxygen (-O-) groups. This is because the carbonyl oxygen has a stronger electronegativity, making it the preferred hydrogen bond acceptor site. After grafting with KH550, the amino groups (-NH_2_) introduced on the Si_3_N_4_ surface can provide two hydrogen atoms to form stable double hydrogen bond structures with the PI carbonyl oxygen, significantly enhancing the interfacial hydrogen bond density, thereby strengthening the organic–inorganic interphase interactions. It is particularly noteworthy that the amine groups (-NH) at the ends of the PI chains, which are not fully imidized, may form a small number of hydrogen bonds with adjacent chain segments. This “end-group effect” is a simulation artifact caused by the simplification of the model. When the polymerization degree of actual materials reaches the hundreds, the proportion of end groups is negligible. However, in the simulation, the limited chain length model often exaggerates the proportion of end groups, leading to an overestimation of the interfacial hydrogen bond density [51]. To address this, the model used in this study was based on a PI chain with a polymerization degree of 15, which has been verified to effectively suppress the end group effect [39], allowing for an accurate analysis of the interfacial bonding mechanism in PI/Si_3_N_4_ composites.

**Figure 13:**
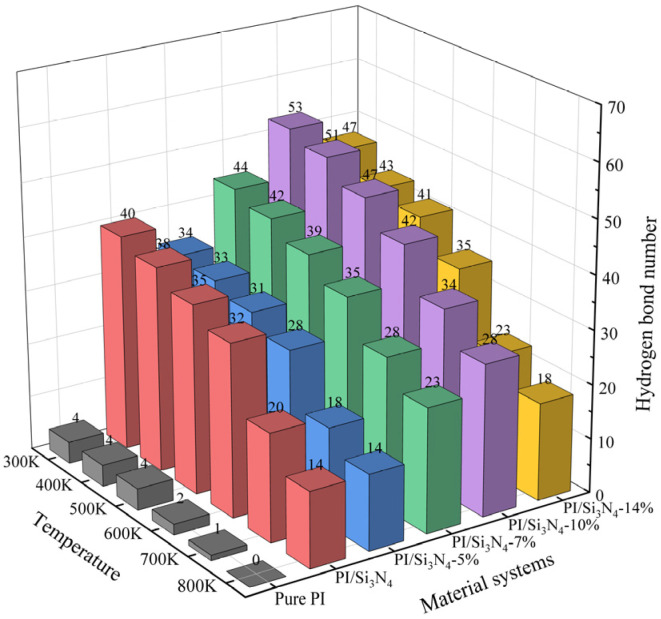
Number of intermolecular hydrogen bonds in different systems from 300 K to 800 K.

Figure 13 summarizes the number of intermolecular hydrogen bonds for each model in the temperature range of 300–800 K. The results indicate that the number of hydrogen bonds generally decreases with increasing temperature, primarily due to the intensified thermal motion of the molecular chains at higher temperatures, which reduces the spatial compatibility between hydrogen bond donors and acceptors, thereby disrupting the existing hydrogen bond network. Notably, in the 600–700 K range, all systems exhibit a sharp decline in the number of hydrogen bonds, which is closely related to the subsequent glass transition temperature and the mechanical property inflection points. In contrast, the hydrogen bond decay rate in the 10% grafting density system is relatively moderate, indicating that its hydrogen bond network in the interfacial region is more stable and can effectively suppress hydrogen bond dissociation at high temperatures.

Comparing the number of hydrogen bonds at the same temperature for the six models, it can be seen that the pure PI system has very few hydrogen bonds, confirming that the model effectively shields the interference of the end-group effect. Except for the 5% grafting density system, the number of hydrogen bonds in all grafted systems is higher than in the unmodified system. Specifically, at 300 K, the systems with grafting densities of 7%, 10%, and 14% showed increases of 10%, 32.5%, and 17.5%, respectively. This enhancement is primarily due to two factors: first, the grafting of the coupling agent increases the hydrogen bond donor density on the Si_3_N_4_ surface, with the amino groups forming a multi-tooth coordination network with PI via the dual hydrogen bond donor characteristic; second, the substitution of hydroxyl groups by amino groups inhibits the self-association of surface hydroxyl groups, converting intramolecular hydrogen bonds into more efficient intermolecular interactions, thereby improving hydrogen bond bonding efficiency. However, at a 5% grafting density, the vertical orientation of the KH550 molecules causes significant steric hindrance, increasing the distance between PI and Si_3_N_4_, which reduces the number of hydrogen bonds by 15%, counteracting the advantage of active sites provided by the grafting. These results reveal the microscopic mechanism of interfacial hydrogen bond formation, providing theoretical support for optimizing the design of composite material interfaces.

It is worth discussing that the nanosecond-scale relaxation simulations in this study are generally sufficient to resolve the formation of local hydrogen bonds in the interfacial region and their average numbers; however, they remain inadequate for fully sampling slower processes, such as the extended rearrangement of hydrogen bond networks or the slow diffusion of chain segments. Therefore, this work focuses on correlating the statistical characteristics of the local interfacial structure at equilibrium—such as the average number of hydrogen bonds, coordination distributions, and radial distribution function peaks—with the material properties. In future work, the simulation duration will be extended to the multi-nanosecond scale to comprehensively explore the complete kinetic pathway of hydrogen bond network formation and to verify the stability of its hierarchical structure and statistical averages over longer time scales.

### 3.5. Calculation of Glass Transition Temperature

Glass transition temperature (*T_g_*) is a characteristic parameter that represents the transition of polymer-based composites from the glassy state to the rubbery state. It is an important indicator for measuring the material’s thermal stability and application temperature range. On a microscopic scale, *T_g_* corresponds to the temperature at which polymer chain segments acquire sufficient thermal energy to overcome the potential energy threshold, marking the critical point of the phase transition from the frozen state to local segmental motion. On a macroscopic scale, it is manifested as a significant change in physical properties such as volume and density around *T_g_*. In this study, based on molecular dynamics simulations, the *T_g_* range of each system was qualitatively determined using the mean square displacement (MSD) method, and its value was accurately calculated using the specific volume–temperature curve method.

**Figure 14:**
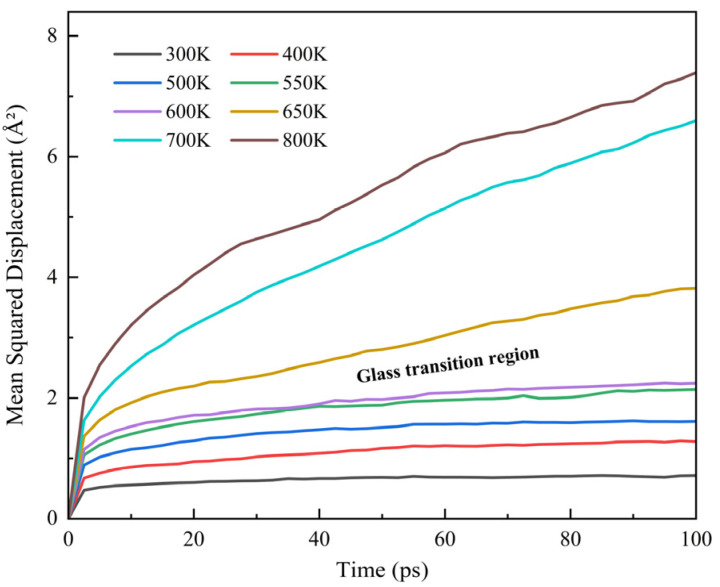
MSD curves of PI at different temperatures.

MSD is a microscopic parameter that characterizes the mobility of polymer chains, and its calculation formula is shown in Equation (5). According to the microscopic mechanism of *T_g_*, the MSD–time curve exhibits a sharp slope change near *T_g_*. Taking the pure PI system as an example, the MSD analysis results in the 300–800 K temperature range (Figure 14) show that when the temperature is below 600 K, the MSD value remains stable below 2 Å, with the slope close to zero, indicating that the molecular chains are in a restricted frozen state. When the temperature exceeds 600 K, the slope of the MSD curve increases significantly, indicating that the molecular chains acquire enough thermal energy to begin local motion. From this, it can be inferred that the predicted *T_g_* range for the pure PI system is 600–650 K.

(5) $$MSD=\frac{1}{3N}\sum_{i=0}^{N-1}\left({\left|r_{i}(t)-r_{i}(0)\right|}^{2}\right)$$

where $r_{i}(t)$ and $r_{i}(0)$ represent the displacement vectors of any atom *i* in the system at time *t* and the initial time, and *N* denotes the total number of atoms in the system.

**Figure 15:**
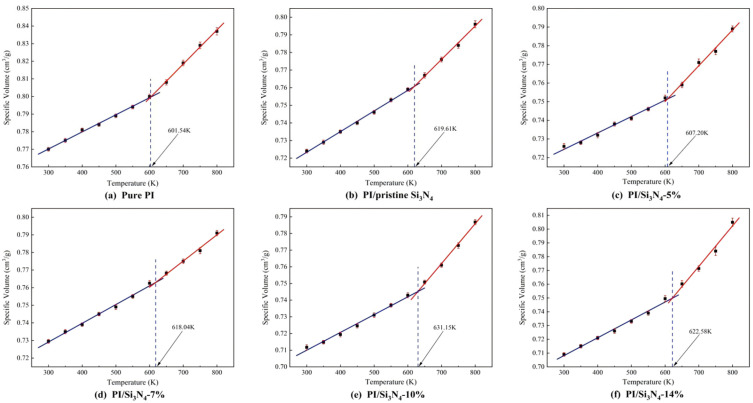
Temperature-specific volume curves of different systems. (**a**) pure PI; (**b**) PI/pristine Si_3_N_4_; (**c**–**f**) PI/Si_3_N_4_ with 5%, 7%, 10%, and 14% KH550 grafting.

To further determine the glass transition temperature, the density of each model at each temperature point in the range of 300 K to 800 K was extracted, and the inverse of the density was used to obtain the specific volume. A temperature-specific volume scatter plot was then constructed, as shown in Figure 15. Linear fitting was performed on the data on both sides of the inflection point, and the x-coordinate of the intersection of the fitted lines corresponds to the *T_g_* value. Table 3 summarizes the simulated *T_g_* values for each system, and the results are consistent with the previously predicted range based on MSD. The simulated *T_g_* value for the pure PI system is 601.5 K, which is close to the experimentally measured *T_g_* value of 575 K reported in the literature [52], validating the reliability of the simulation method.

The data in Table 3 show that the *T_g_* values of the five PI/Si_3_N_4_ composite systems are all higher than that of the pure PI system. This can be explained by the free volume theory in Section 3.2: the rigid nanoparticles occupy part of the free volume of polyimide, compressing the molecular chain’s mobility and thus restricting segmental motion and improving thermal stability. Among the composite systems, the 10% grafting density system shows the most significant *T_g_* increase, rising by about 30 K compared to the pure PI system. This indicates that the hydrogen bond network in this system is more intact and the interfacial interactions are stronger, requiring the PI molecular chains to overcome a higher potential energy barrier to move. In contrast, the 5% grafting density system, due to the isolated distribution of the coupling agent on the nanoparticle surface, has a looser interfacial bonding and a discontinuous hydrogen bond network, resulting in weaker confinement of the PI segments. The *T_g_* of this system increases by only about 6 K compared to the pure PI system, which is consistent with the hydrogen bond analysis results in Section 3.4. In summary, the thermal stability of polymer composites is collaboratively regulated by microstructural parameters such as interfacial binding energy, hydrogen bond quantity, and free volume fraction. Therefore, bridging the microstructure in materials with macroscopic properties has significant theoretical importance for the design of high-stability composites.

### 3.6. Calculation of Thermal Conductivity

Thermal conductivity is an important parameter for evaluating the thermal performance of materials. Increasing the thermal conductivity can enhance the heat dissipation ability of polyimide materials, thereby lowering the operating temperature of equipment. The current methods for calculating thermal conductivity at the microscopic scale mainly include the non-equilibrium molecular dynamics (NEMD) method based on Fourier’s law and the equilibrium molecular dynamics (EMD) method based on the Green–Kubo formula [53,54]. Given the drawbacks of the EMD method, such as low computational efficiency and poor convergence, this study chooses the reverse perturbation non-equilibrium molecular dynamics (RNEMD) method within the NEMD framework, which is closer to experimental conditions [55].

**Figure 16:**
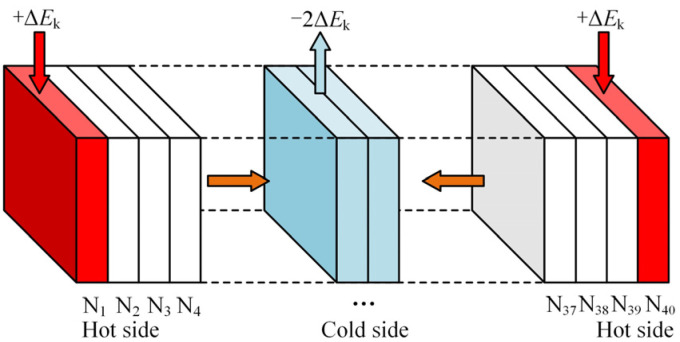
Schematic diagram of RNEMD principle.

The principle of the RNEMD method is shown in Figure 16: The model is divided into 40 layers along the z-axis, with the two ends representing high-temperature regions (heat source layers) and the central region representing the low-temperature region (cold source layer). The temperature of the material is the average temperature of each layer in the model. Within a set time interval, an energy flux is applied by exchanging the most energetic particles in the cold source layer with the least energetic particles in the heat source layer, driving the energy transfer from the two heat sources to the central cold source, until a stable temperature gradient and constant energy flux are gradually established in the system. Finally, the thermal conductivity is calculated based on Fourier’s law, as shown in Equations (6) and (7):

(6) $$\lambda =-\frac{J}{\;dT/dz}$$

(7) $$J=\frac{1}{2A}\frac{\bar{\Delta E}}{\Delta t}$$

where J is the energy flux in the z-direction, dT/dz is the temperature gradient, λ is the thermal conductivity, *A* is the cross-sectional area perpendicular to the flux direction, Δ*E* is the amount of exchanged energy, and Δ*t* is the exchange time.

**Figure 17:**
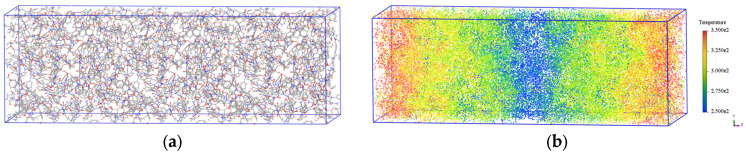
Simulation diagram of thermal conductivity in the pure PI system. (**a**) Supercell model; (**b**) spatial temperature distribution.

**Figure 18:**
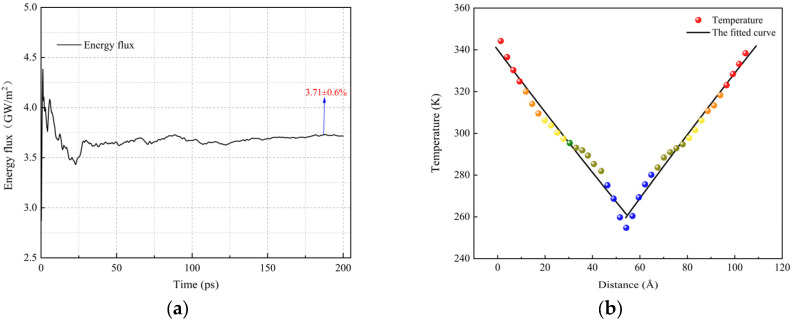
Energy flux and temperature gradient in the pure PI system. (**a**) Energy flux versus time curve; (**b**) steady-state temperature gradient.

Taking the thermal conductivity calculation of pure PI at 300 K as an example, the original model in Figure 5 was first expanded by three times along the *Z*-axis to construct a supercell (Figure 17a), in order to minimize the finite size effects on the calculation accuracy. Then, under the NVT ensemble, the system was equilibrated by performing 500 kinetic energy exchanges and using a heat bath temperature control method, achieving a steady-state temperature distribution after applying the energy flux (Figure 17b). Finally, the system was switched to the NVE ensemble, and energy flux and temperature gradient data were collected, as shown in Figure 18. In Figure 18a, the energy flux stabilizes in the last 50 ps of the simulation, and the average value of this segment is taken as the final data. In Figure 18b, the temperature distribution on both sides of the cold source is clearly symmetric, and the temperature gradient is obtained by linearly fitting the temperatures of each layer. By substituting the relevant data into Equation (6), the thermal conductivity of pure PI at 300 K is found to be 0.204 W/(m·K), which is close to the experimental value of 0.15–0.2 W/(m·K) [56], validating the reliability of the method. The thermal conductivities of all systems calculated using this method are summarized in Table 4.

**Figure 19:**
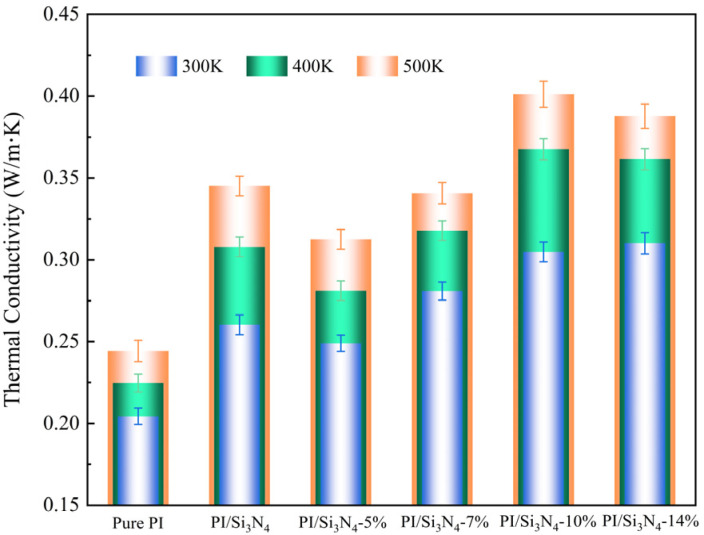
Temperature response characteristics of thermal conductivity in different systems.

The data in Table 4 show that the thermal conductivity of the composite systems with different grafting densities of Si_3_N_4_ is increased by 27.4%, 22.1%, 37.7%, 49.5%, and 51%, respectively, compared to pure PI. This improvement is mainly attributed to the intrinsic high thermal conductivity of Si_3_N_4_ and the interfacial effects induced by grafting modification: On one hand, the grafted coupling agents enhance the interfacial bonding strength between the filler and the matrix, improving the stacking order of the molecular chains, which effectively reduces interfacial phonon scattering and extends the average free path. On the other hand, the increase in grafting density promotes the increase in the density of the interfacial hydrogen bond network, which expands the phonon transport path, further reducing the interfacial thermal resistance and improving thermal conductivity. Figure 19 reveals the temperature-dependent thermal conductivity characteristics of each system. It can be seen that the thermal conductivity increases with temperature, and the increase in the composite systems is significantly higher than that of pure PI. This is because the grafted composite systems exhibit cooperative motion of the matrix and coupling agent chains at high temperatures, enhancing the interfacial phonon coupling effect and further promoting heat transfer across the interface. However, when the grafting density increases to 14%, excessive grafting forms a thicker organic layer, which, under thermal disturbance, can easily lead to interfacial structural disorder, disrupting the thermal conduction network and increasing phonon scattering, resulting in a decline in the thermal conductivity increase. In conclusion, silane coupling agent grafting, as an interfacial modification method, can effectively enhance the thermal conductivity of composite materials, but the grafting density must be carefully controlled to avoid performance degradation caused by interfacial structure imbalance.

### 3.7. Calculation of Mechanical Moduli

The mechanical modulus is an important parameter for measuring the stress–strain response, and its value directly determines the material’s resistance to deformation and structural rigidity during elastic deformation. In this study, the static constant strain method was used to calculate the mechanical modulus of PI and its composites. The calculation principle involves applying small deformations along the xx, yy, zz, yz, xz, and xy planes to the model in a balanced state. The stiffness matrix *C_ij_* is obtained based on the model’s response to the deformation, and the matrix elements are calculated using the following formula:

(8) $$C_{ij}=\frac{1}{V}\cdot \frac{\partial^{2}U}{\partial \varepsilon_{i}\partial \varepsilon_{j}}=\frac{\partial \sigma_{i}}{\partial \varepsilon_{j}}=\frac{\sigma_{+}-\sigma_{-}}{2\varepsilon_{j}}$$

where U is the potential energy, σ is the stress, which is the first derivative of the potential energy with respect to strain per unit volume, “+” denotes tension, “−” denotes compression, and ε represents the strain.

The calculation revealed that the stiffness matrix is symmetric, allowing PI to be treated as an isotropic material. The Lame constants λ and μ can be obtained from the stiffness matrix as follows:

(9) $$\lambda =\frac{1}{3}\left(C_{11}+C_{22}+C_{33}\right)-\frac{2}{3}\left(C_{44}+C_{55}+C_{66}\right)$$

(10) $$\mu =\frac{1}{3}\left(C_{44}+C_{55}+C_{66}\right)$$

From this, the material’s Young’s modulus and shear modulus can be calculated, as shown in Equations (11) and (12):

(11) $$E=\mu \cdot \frac{3\lambda +2\mu }{\lambda +\mu }$$

(12) G=μ

**Figure 20:**
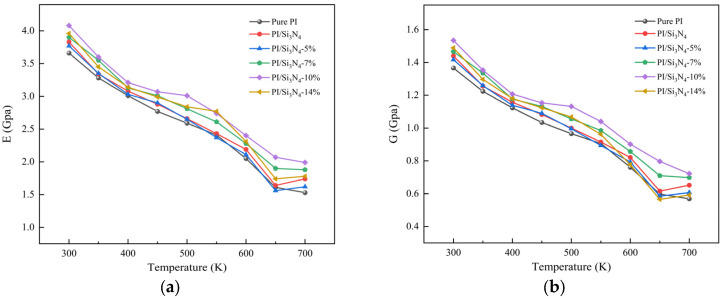
Temperature-dependent curves of mechanical moduli. (**a**) Young’s modulus; (**b**) shear modulus.

The curves of Young’s modulus and shear modulus as a function of temperature, with a step size of 50 K, are shown in Figure 20. The simulated Young’s modulus for pure PI is 3.66 GPa, which falls within the range of the experimental values of 3.2–3.8 GPa reported in the literature [57,58], validating the reliability of the simulation method. The results show that both the Young’s modulus and shear modulus of all systems gradually decrease with increasing temperature, with the most significant decrease occurring in the 600–650 K range, which is consistent with the *T_g_* values calculated in Table 3. This change is primarily due to the expansion of free volume and the rupture of interfacial hydrogen bonds caused by the temperature increase, thereby weakening intermolecular interactions. As the temperature approaches *T_g_*, the PI matrix transitions from the rigid glassy state to the flexible rubbery state, enhancing the molecular chain movement and causing a sharp drop in the overall mechanical modulus.

In the wide temperature range of 300–700 K, the Young’s modulus and shear modulus of the 10% grafting density PI/Si_3_N_4_ composite system are significantly superior to those of the other systems, with the most noticeable improvement in mechanical properties, while the low-grafting-density and unmodified systems show limited improvement compared to pure PI. This phenomenon can be attributed to three factors: First, as noted in Section 3.6, the thermal conductivity of the 10% grafting system is approximately 50% higher than that of pure PI, significantly enhancing the material’s thermal dissipation efficiency and effectively suppressing structural damage caused by thermal accumulation. Second, the low-grafting-density systems exhibit stress concentration and micropore defects at the interface, weakening the overall mechanical performance, while the high-grafting-density systems fill these interface defects by constructing a dense hydrogen bond network, allowing for efficient stress transfer. Third, Figure 6 and Figure 7 previously confirmed that the 10% grafting density system has the strongest interfacial binding energy and the lowest free volume fraction, which effectively suppresses the relaxation of molecular chain segments and the expansion of free volume at high temperatures, thus maintaining excellent mechanical performance across a wide temperature range.

It is particularly worth noting that material property metrics obtained from molecular dynamics simulations may deviate to some extent from experimental measurements. On the one hand, such differences arise from systematic errors during experiments, including fluctuations in ambient temperature and humidity and limitations in instrument precision [59]; on the other hand, they reflect the idealized boundary conditions adopted in the simulations and the simplified model that considers only a single Si_3_N_4_ nanoparticle and PI chains with a degree of polymerization of 15. While this simplification facilitates isolating the influence of silane grafting density on interfacial properties under a controlled model size and sampling protocol, it may underestimate nanoparticle agglomeration and the structural heterogeneity present in real materials [60]. To more comprehensively evaluate these effects, future work will extend the current framework to systems comprising multiple nanoparticles and longer polymer chains.

Nevertheless, molecular dynamics simulations retain distinct advantages for elucidating structure–property relationships that link microstructure to macroscopic performance. In this study, PI/Si_3_N_4_ composite models with varying silane grafting densities were constructed to systematically clarify how interfacial modification regulates thermal and mechanical properties. The simulation outcomes provide a predictive basis for subsequent experiments and underpin a “computational design–experimental validation” co-optimization framework. On this basis, future work will involve fabrication of KH550-modified PI/Si_3_N_4_ composite films and characterization of their thermal and mechanical responses using laser flash analysis, differential scanning calorimetry, and dynamic mechanical analysis. Direct comparison between experimental measurements and simulation predictions will enable quantitative assessment of model fidelity and further refine the “computational design–experimental validation” framework, thereby establishing a robust foundation for cross-scale correlation analysis and property-oriented control of high-performance polymer–matrix composites.

## 4. Conclusions

In this study, molecular dynamics simulations were employed to systematically investigate the effects of nano-Si_3_N_4_ doping and varying silane coupling agent grafting ratios on the microstructure and macroscopic properties of PI-based composites. Covering a wide temperature range of 300–700 K and utilizing multiscale characterization techniques including *E_int_*, FFV, RDF, and hydrogen bond network analysis, the study elucidated the regulatory mechanisms of interfacial modification on thermal and mechanical properties and established quantitative structure–property relationships. The main findings are summarized as follows:

(1) Silane grafting significantly enhanced the interfacial compatibility between nano-Si_3_N_4_ and the PI matrix. At a 10% grafting density, the non-bonded interaction energy increases by 18.4% relative to the unmodified system, and the number of hydrogen bonds rises by 32.5%. RDF analysis reveals high-density coordination between the terminal amino groups of the coupling agent and the carbonyl groups of PI, further confirming the formation of a compact and stable hydrogen bond network at the interface.
(2) The incorporation of nano-Si_3_N_4_ compresses the composite’s free volume. At a 10% grafting density, FFV attains a minimum (18.13%), thereby constraining the segmental thermal motion of PI chains and increasing *T_g_* by approximately 30 K relative to pure PI. An appropriate grafting level maintains a robust hydrogen bond network at elevated temperatures, suppresses thermally induced volumetric expansion, and confers excellent thermal stability across a broad temperature range.
(3) Grafting modification substantially enhances the composite’s thermal conductivity. At a 10% grafting density, the thermal conductivity reaches 0.305 W/(m·K), representing a 49.5% increase relative to pure PI. The strengthened interfacial adhesion and the formation of a dense hydrogen bond network effectively reduce interfacial thermal resistance and suppress phonon scattering, thereby markedly improving heat-transfer efficiency.
(4) Interfacial modification markedly increases interfacial binding energy and optimizes stress-transfer pathways, thereby significantly improving the composite’s mechanical properties. Across 300–700 K, the 10% grafted system consistently exhibits the highest Young’s modulus and shear modulus, with pronounced enhancements over pure PI.

In conclusion, a 10% KH550 grafting density emerges as a practical optimum for high-temperature, high-reliability PI/Si_3_N_4_ composites, providing a quantitative design rule for interfacially engineered PI films used in aerospace thermal/electrical insulation and flexible electronics, with the potential to markedly enhance device-level thermal management and mechanical integrity. It should be noted that the PI/Si_3_N_4_ interface considered here is dominated by noncovalent interactions; introducing reactive functionalities to promote covalent coupling could modify interfacial behavior and shift the optimal grafting level, so the quantitative conclusions are most applicable to analogous nonreactive polymer/inorganic systems. In addition, the present model—single Si_3_N_4_ nanoparticle, relatively short PI chains, and idealized conditions—entails limitations. Future work will extend to multiparticle systems, broader chain length distributions, and more realistic operating environments via multi-scale simulations, and will be integrated with experimental synthesis and device-level testing to refine cross-scale design and application guidance.

## Figures and Tables

**Table 1:** Average length of the silane coupling agent.

Surface Grafting Ratio	Average Length of the Coupling Agent
(Initial length)	5.35 Å
KH550/Si_3_N_4_-5%	4.94 Å
KH550/Si_3_N_4_-7%	4.45 Å
KH550/Si_3_N_4_-10%	4.06 Å
KH550/Si_3_N_4_-14%	4.79 Å

**Table 2:** Volume fraction of free volume for each system at 300 K.

System	*V_Free_*/nm^3^	*V_Occupied_*/nm^3^	*FFV*/%
Pure PI	9.421	34.748	21.33
PI/Si_3_N_4_	9.128	37.994	19.37
PI/Si_3_N_4_-5%	9.508	38.129	19.96
PI/Si_3_N_4_-7%	9.298	38.779	19.34
PI/Si_3_N_4_-10%	8.506	38.409	18.13
PI/Si_3_N_4_-14%	8.747	38.383	18.56

**Table 3:** Simulated glass transition temperatures of different systems.

System	Glass Transition Temperature
Pure PI	601.5 K
PI/Si_3_N_4_	619.6 K
PI/Si_3_N_4_-5%	607.2 K
PI/Si_3_N_4_-7%	618.0 K
PI/Si_3_N_4_-10%	631.2 K
PI/Si_3_N_4_-14%	622.6 K

**Table 4:** Simulated thermal conductivity of different systems.

System	Thermal Conductivity [W/(m·K)]		
	300 K	400 K	500 K
Pure PI	0.204	0.225	0.244
PI/Si_3_N_4_	0.260	0.308	0.345
PI/Si_3_N_4_-5%	0.249	0.281	0.313
PI/Si_3_N_4_-7%	0.281	0.318	0.341
PI/Si_3_N_4_-10%	0.305	0.368	0.401
PI/Si_3_N_4_-14%	0.308	0.362	0.388

## Data Availability

The original contributions presented in this study are included in the article. Further inquiries can be directed to the corresponding author.

## Abbreviations

The following abbreviations are used in this manuscript:

MD	Molecular Dynamics Simulation
PI/Si3N4	Polyimide/unmodified nano-silicon nitride composite system
PI/Si3N4-5%	Polyimide/nano-silicon nitride composite system with 5% surface silane grafting
PI/Si3N4-7%	Polyimide/nano-silicon nitride composite system with 7% surface silane grafting
PI/Si3N4-10%	Polyimide/nano-silicon nitride composite system with 10% surface silane grafting
PI/Si3N4-14%	Polyimide/nano-silicon nitride composite system with 14% surface silane grafting
CED	Cohesive Energy Density
FFV	Fractional Free Volume
RDF	Radial Distribution Function
MSD	Mean Square Displacement
Tg	Glass Transition Temperature
RNEMD	Reverse Non-Equilibrium Molecular Dynamics
